# Announcement: Cost D8 and ESF Workshop on Biological and Medicinal Aspects of Metal Speciation

**DOI:** 10.1155/MBD.1997.294

**Published:** 1997


					COST D8 AND ESF WORKSHOP
ON BIOLOGICAL AND MEDICINAL
ASPECTS OF METAL SPECIATION
23-25 August 1998,
Szeged, Hungary
For further information contact"
Professor Tamas Kiss
Department of Inorganic and Analytical Chemistry
Jozsef Attila University
PO Box 440
H-6701 Szeged, HUNGARY
E-mail"
Tel" +36 62 454337
Fax" +36 62 312505
tkiss@chem.u-szeged,hu
294

				

